# Managing Risk & Seeking Dignity: Working‐Class Perceptions of University in London, Rochdale & Morecambe

**DOI:** 10.1111/1468-4446.70021

**Published:** 2025-07-26

**Authors:** Amit Singh

**Affiliations:** ^1^ Department of Sociology University of Manchester Manchester UK

**Keywords:** class, debt, dignity, risk, university, working‐class

## Abstract

This paper examines how working‐class young people enroled at college in London, Rochdale and Morecambe perceive of university. It argues that university represents a great risk, associated with high levels of debt, which does deter some students, but at the same time, university is imagined as a meaningful vehicle for dignity and respect, which students place greater value on than the prospect of benefitting from the so‐called “graduate premium”. Broadly, then, it argues that the desire to attend university is predicated on three factors: the calculation of risk versus reward, the “migrant effect” for the children of migrants or those who migrated directly, and thirdly, the pursuit of dignity and respect.

## 1 Introduction

Despite a questionable link between social mobility and university attendance, university is often regarded as an important vehicle for social mobility (Boliver 2017). This is particularly the case in the context of an expanding university sector that now accepts working‐class students in increasing numbers, which is widely lauded as a positive development that is encouraged by policy‐makers (e.g. Department for Education 2016; Gov 2021). Though relatedly much has been written about working‐class experiences of university (Reay et al. 2009; Boliver 2013; Reay 2017; Gamsu et al. 2019; Donnelly and Gamsu 2020) and transitions from university to the labour market (Bathmaker et al. 2016; Bradley and Waller 2017; Bathmaker 2021; Ingram et al. 2023) less attention has been paid to working‐class teenagers' perceptions of attending (see Reay et al. 2001, Singh 2024 for exceptions) particularly in a context of tuition fees tripling since 2010 (see Callender and Mason 2017, Harrison 2018, Harris et al. 2020 for notable exceptions). Working‐class youth contend with these decisions against the backdrop of high youth unemployment and a diminishing “graduate premium” (the supposed increased wages associated with a degree), especially outside of London (Stansbury et al. 2023), where an increasing number of graduates work non‐graduate jobs (Xu 2023).

Responding to this context, this paper offers a qualitative exploration of how working‐class young people (16+) in London, Rochdale, and Morecambe perceive of university as both carrying great risk in the form of debt, but also as a means to garner dignity otherwise denied, in a context where young people see few tangible alternatives. As will be argued, though perceptions around debt do turn some students away from a potential degree, the allure of university ‐ though tied to material benefit ‐ is largely about the pursuit of dignity, given the social and cultural work done over previous decades to associate a university degree with respectability and middle‐classness (see Sennett and Cobb 2023; Savage 2007).

### 1.1 Working‐Class Students & University

Before the onset of deindustrialisation and a turn towards a flexible service economy, it was commonplace for working‐class young people to leave school at 16 to find readily available working‐class jobs (Furlong and Cartmel 2007). This was the focus of Paul Willis' *Learning to Labou*r, an exploration of how working‐class boys get working‐class jobs in Wolverhampton. With pre‐ordained routes into work, Willis found that these “lads” had little regard for education, as he aptly described “the ‘tumble’ out of school” predicated on “the prospect of money and cultural membership amongst ‘real men’” (Willis 1977/1981, 100). Yet, the onset of deindustrialisation and the economic restructuring that accompanied it resulted in a drastic decline in the sort of jobs that Willis' “lads” undertook, prompting research on how young men living in deindustrialised areas have responded to these contexts (e.g. Nayak 2003; Ward 2017; Gater 2023). Relatedly, it is worth noting that previously available industrial work was largely masculine labour, as a major impact of deindustrialisation was the increasing participation of working‐class women in the labour force, albeit typically in poorly paid, insecure work (McDowell and Massey 1984; Walkerdine et al. 2001).

In light of these shifts, young people now spend extended time in post‐16 education. A significant part of this trend has been the rapid expansion of higher education provision in the UK (and beyond). At the time of Willis' research ‐ and until the 1990s ‐ university was primarily the preserve of the middle and upper classes. In the 1950s, only 3% of the university‐aged population went to university, which grew to around 15% by 1988 (Bathmaker et al. 2013; Evans 2023, 212), then ballooned to 50.2% by the 2017‐2018 academic cycle.^1^ The 1992 Further and Higher Education Act, which awarded university status to what were once known as Polytechnics was central to the expansion of the higher education sector, as the number of universities in the UK grew from 31 to 134 (Boliver 2013).

Accordingly, working‐class students now attend university in increasing numbers, leading to an increase in academic research focusing on working‐class experiences at university, including work exploring how working‐class students cope within middle‐class and elite institutions (Reay et al. 2001; Reay et al. 2009; Abrahams and Ingram 2013). For instance, the paired peers project, a longitudinal comparative study of students at the University of Bristol and the University of West England extensively examined working‐class attitudes towards university, experiences of university and the role of class, as well as geography, on graduate outcomes (see Bathmaker et al. 2016; Ingram et al. 2023). One implication of the expansion of universities has been debate about whether the graduate premium has diminished or not. Here, it is worth flagging two concerns for working‐class young people. Firstly, they are more likely to enter post‐1992 universities, where drop‐out rates are higher, and they achieve degrees that can be undervalued (Boliver 2013; Reay 2017) as many recruiters target only the most prestigious universities (Wakeling and Savage 2015). The second concern is that middle and upper‐class graduates dominate the graduate labour market as a result of their ability to distinguish themselves from working‐class or lower middle‐class candidates through possessing the relevant social and cultural capital, including the relevant social networks to exploit, the ability to undertake unpaid internships and knowledge of the “right” extra‐curricular activities (Bathmaker et al. 2013; Rivera 2015; Abrahams 2017; Ashley and Empson 2017). Drawing upon Bourdieu (1986), this paper is concerned with working‐class young people who lack economic, cultural and social capital, which hinders their ability to plan for the future and navigate middle‐class and elite institutions such as schools and universities. Broadly, social capital can be understood as social networks (e.g. parents and their social circles, which, as Hardie (2022) has shown, alter young people's inclination towards or away from work and university). Economic capital is access to financial resources (pocket money, money to pay tuition fees and accommodation, supplement loans, or a home to return to on graduation). Cultural capital is an inherited understanding of how social institutions work and how to behave in different social contexts. Deployment of cultural and social capital is increasingly crucial to navigating a crowded labour market (Brown 2013), where a degree alone is insufficient to secure a graduate job (again, see Bathmaker et al. 2013; Abrahams 2017; Ingram et al. 2023).

Significantly, university was free until 1998, when New Labour introduced £1000 a year tuition fees, followed by the Higher Education Act in 2004, which tripled fees to £3000 per year before fees were tripled again to £9000 in 2010, a nine‐fold increase in just 12 years (Deardren et al. 2011) before rising to £9535 in November 2024. Accordingly, there has been a growing body of work exploring how the notion of debt figures in young people's future planning towards or away from university. There has, for instance, been work focusing on the extent to which debt has put working‐class people off attending university (Callender and Mason 2017; Callender and de Gayardon 2021; Callender and Davis 2024). Strands of this work have explored the psychological burden debt places upon young people (Harris et al. 2020; Callender and Davis 2024), as well as the risk management strategies young people deploy, which, for Harrison, involve insurance against the alternative, facing unemployment in an uncertain youth labour market (Harrison 2018).

Whilst university attendance is often used as a stand‐in for social mobility (e.g. Sewell et al. 2021; Gov 2021), the link between university and social mobility has weakened (Boliver 2017). Significantly, there are regional differences between graduate outcomes as London remains home to a higher concentration of graduate jobs (Xu 2023, Davies and Donnelly 2023). In turn, there is an oft‐discussed “London escalator”, wherein it is assumed that working‐class Londoners have greater chances of social mobility. This narrative has been widely critiqued (e.g. Friedman and Macmillan 2017; Ingram et al. 2023; Yu et al. 2024) as though London is home to a higher proportion of high‐skilled graduate job opportunities, these disproportionately go to those from affluent backgrounds, which reproduces class inequalities within the capital (Ingram et al. 2023, see also Yu et al. 2024). Still, Ingram and colleagues found that having a home in London, even for working‐class students, can act as “an enabler for graduates”, given the proximity to potential graduate destinations in London, not least because this enables people to perform unpaid internships or low‐paid work which would be impossible for working‐class young people forced to privately rent in the capital (Ingram et al. 2023, 50) such as those from Rochdale or Morecambe. In part responding to this policy climate, this paper then examines how working‐class young people across geographic locations, racial groups and gender, understand the potential transition to university, particularly in the context of rapid changes in the labour market and the significant increase in tuition fees.

## 2 Methods

The data presented in this paper derives from a wider qualitative research project exploring how working‐class young people (16+) in London, Rochdale and Morecambe imagine their future transitions out of college and into higher education and/or the labour market. It explored these processes across four field sites: one sixth‐form college in London (Park Walk), one sixth‐form college (Newgate College) and one vocational college (Woodhouse College) in Rochdale, and one vocational college in Morecambe (MTC). Students at Park Walk and Newgate College were undertaking A‐Levels, whereas the students at MTC and Woodhouse College were undertaking various vocational qualifications such as T‐Levels and Level 1, 2 and 3 diplomas. A Level 1 Diploma is for those who have failed a large number of GCSEs and represents a low‐level GCSE, a Level 2 diploma is a higher level GCSE equivalent (both of these are taken over 1 year each) and a Level 3 diploma (usually over 2‐years) can (but not always) represent A‐Level equivalency and offer a route into university (sometimes requiring an additional foundation year at university).

The names of all colleges and participants have been pseudonymized. In total, I interviewed 60 young people across the sites with a relatively even gender split. The Morecambe sample was exclusively white, reflecting the demographics of the area, whilst the London and Rochdale samples were more reflectively diverse. Park Walk is located in London in one of the UK's most deprived boroughs, drawing in students from the local population, many of whom have been excluded from more academically competitive local academies. Newgate College and Woodhouse College are in Rochdale, a former mill town in Greater Manchester that grew out of the colonial trade in cotton but, due to the processes of deindustrlisation and economic restructuring, is now regarded as an in decline town (Pike et al. 2016). Morecambe is a coastal town 65 miles north of Rochdale that was once a prominent tourist destination for industrial workers in the North of England but today is one of the most deprived places in the UK (McDowell and Bonner‐Thompson 2020). These areas were selected due to their centrality to wider narratives that map inequality onto geography and make much of how working‐class white students in the North and in coastal towns are less likely to go to university than their ethnic minority counterparts in London (e.g. Sewell et al. 2021; Gov 2021). I gained access to the college through a combination of identifying relevant gatekeepers, pre‐existing links and cold‐calling colleges. Staff at the college then facilitated my research, putting me in touch with course leads to identify participants.

Students undertook a demographic survey to measure social class, followed by a semi‐structured interview that lasted between 45 and 75 min, all of which were conducted at the respective college the student attended. As outlined elsewhere (see Singh 2024) the demographic survey followed other approaches to class categorisation (e.g. Friedman and Laurison 2019) by determining parental occupations and level of qualifications, whether students were eligible for free school meals or not (34 out of 60 were), whether students lived in social housing, and how students identified their own class. It was designed to measure the levels of social, cultural and economic capital students had access to. Out of 60 students, 28 students actively wanted to attend university; All of those in London and all students at Newgate College (reflecting how these were A‐Level focused colleges), whilst only two out of 26 at MTC did, and a further 10 out of 18 at Woodhouse College.

### 2.1 Perceptions of Debt: “Risk Versus Reward”

Responding to the aforementioned emerging literature on how rising fees have influenced young people's perceptions of university, and inclination to attend university, this section addresses how young people discuss the notion of debt, largely through “debt aversion” (Baum and Schwartz 2013; Callender and Mason 2017), as well as deploying strategies to mitigate against debt, including staying at home (see Donnelly and Gamsu 2020, Harris et al. 2020) and seeking out part‐time employment alongside studying. Of those involved in the broader study this paper emerges from only seven students had a parent who had been to university; two had mothers who had attended university as mature students to attain greater financial stability (Singh 2024); one had a parent who attended university but is long‐term unemployed; and four young people who were not born in the UK had a parent who had graduated in their country of origin but now, as is typical of those performing downward social mobility, works a non‐graduate job in the UK (see Modood 2004; Reay 2017). All other students were prospective first‐generation university students with no cultural capital (Bourdieu 1986) to inherit in the form of tacit knowledge about what university would entail, how to apply, or how tuition fees and loans work. The latter point was of particular concern to many students, who associated university with risk based on their perceptions of debt, which has been shown to be a particular concern to ethnic minorities and working‐class young people (Callender and Mason 2017). This was apparent for Maria and Liam, two white working‐class students on free school meals who are studying for a Level 2 in Art in Morecambe.

Maria I swear like… If you get student loans and then you go to uni, sometimes people kind of find jobs and then you're just in debt until you're 50. It's a bit stupid though, like, you're paying all this money to get an education, to get a career that pays good, but then by the time you get your career that pays good, you're just paying back. And then, what, by the time you've actually paid off that debt, how much more are you going to even want to work in that job?

Liam I have considered uni, but it would simply cost too much to attend… I'm pretty sure it costs money to go to university and if you don't have the money then you'll be in debt. And I wouldn't like to be in debt. Because then, well I mean apparently they don't start taking the money off you until you've started making enough, but I would like the idea to know that I am not in debt to someone or something.

Firstly, both students are undertaking Level 2 qualifications, so they would not be eligible to apply for university upon completion of these courses, as they would have to enrol on a Level 3 qualification first. Irrespective, both are deterred by the prospect of debt more than having to complete further qualifications, as at different stages, both considered the prospect of an art degree before dismissing it. On completing their Level 2 qualification, both planned to find local work rather than defer earnings and accrue debt. These sentiments were echoed by Bilal and Arif, two British‐Pakistani boys on free school meals who are studying barbering at Woodhouse College in Rochdale. In contrast to Liam and Maria, they passed their maths and English GCSEs and could have done A‐Levels if they had wanted to.

Bilal I don't like the academic path, going to university and stuff. I feel like it's a big waste of time. When you come out, there's people that come out of university, say for example they do computer science, and I know a few people, like about 4 or 5, they still can't find a job. It's like you went through all of that for a piece of paper that you can't get a job with. You know what I mean? You're wasting money, you're going in debt for nothing.

Arif I think the same, you know, I just think the education system is built off older times. Back in the day the demand for jobs was high. So if you went to uni, you got the degree, you did the qualifications, you had the skills and everything, you most likely get hired 80%. Now you lucky to get hired.

Bilal and Arif are dissuaded from attending university because of the debt and a related fear of ending up in what Brown describes as the “opportunity trap”. a situation wherein young people are told they must go to university to gain professional middle‐class employment, resulting in a situation where there are nowhere near enough graduate jobs to meet demand (Brown 2003). Partially, this resulted from having direct experience with family members and friends who had been to university but had not been rewarded with a graduate job despite the debt they had taken on. Crucially, both work part‐time as barbers whilst studying, making money each week, which they know would have been foregone if they were to pursue university, something that figured in both of their perceptions of university. Bilal went on to argue;

Bilal With university and higher college and stuff, it's a long time, it's almost a decade that you're in education. And then obviously you're wasting money, you're going in debt. I want it to be the opposite, I want to be making money.

Bilal and Arif were not alone in situating attending university as deferring earnings. Gemma, for instance, a marketing apprentice at CTC in Morecambe, was similarly put off from university for this reason, seeing university as costing money and foregoing earnings;

Gemma I'd just rather be in the workplace learning, getting my hands dirty, than get all these other qualifications that I don't need… My goal after this course is to travel, so I feel like uni would take up all my time and my money.

Gemma contrasts the perceived practicality of getting her “hands dirty” at college with the supposed impracticality of a university degree, which she suggests lacks intrinsic real‐world value. This resonates with how the “lads” in Willis' Learning to Labour or the working‐class adults interviewed by Sennett and Cobb in The Hidden Injuries of Class perceived work and education. For example, Frank Rissario, interviewed by Sennett and Cobb, disdained “educated white‐collar work”, reflecting, “these jobs aren't real work where you make something ‐ it's just pushing papers” (Sennett and Cobb 2023, 21). As an apprentice, Gemma earns £5.21 an hour (below the national minimum wage of £8.21 for 18‐20 year‐olds), which contrasts with not being paid for doing A‐Levels or then for going to university, which limits your immediate earning potential due to the requirements to go to class, do coursework etc. For Bilal, Arif and Gemma, university is not seen as a worthwhile investment, though the debt is often couched in these terms (see Harris et al. 2020), as they see going to university as inherently risky, time‐consuming and possibly even pointless when considering university as being purely about financial calculus relating to return on investment.

Other students were worried about debt but still had reluctant plans to apply for university, even if they also expressed “debt aversion”, an anxiety about taking on excessive debt (Callender and Mason 2017; Baum and Schwartz 2013). For instance, Dean, a white working‐class eSports student on free school meals in Rochdale, considered university as a “risk versus reward” calculation. Dean's mum is currently unemployed and his Dad works part‐time in a local snooker hall. Neither attended university or college. Hence, they had no tacit knowledge to pass on to him. Still, both actively encouraged him to consider university as his next step out of college, challenging the problematic argument that working‐class parents have low aspirations for their children (see Allen 2014 for critique).

Dean They [Dean’s parents] have been pushing me a lot further towards it [university], but also reminding me about risk versus reward, knowing that I would get a degree, I'd be able to get into a better job with it, but also knowing that I would be going into the world with that debt and so I'm very split between going into full‐time work and going towards uni… If I went to uni I'd be in at least part time work being able to, a bit of money both for accommodation.

Dean is in the process of applying for part‐time jobs in Greater Manchester, having had interviews at a number of local fast‐food chains in the area. He is also completing his application to university to study eSports at university in the North of England as his goal is to eventually work in the growing eSports industry, which Dean believes requires a degree as a minimum barrier to entry.

Amit Do you think it's important to go uni to get a job in the industry?

Dean I believe so yes, they are looking for really high qualified applicants and obviously I'll have more of a chance if I have a uni degree behind me. But I think about the debt that I can end up with in student loans and what I'd have to do to pay off that in the future. I hate being in debt to anyone.

On the one hand, Dean is put off from attending university by the potential debt and the structuring of student loan repayments. Intuitively, Dean's strong aversion to taking on debt makes sense, as though the student loan is often presented as necessary, or not like other forms of debt (see Harris et al. 2020), it is still borrowing a very large sum of money. This reflects a rejection of the widely assumed logic that the loans are manageable for young people, so should not be thought of as debt, something interrogated by Harris et al. (Ibid) in their exploration of young people's anxiety over debt. On the other hand, he sees attending university as necessary to fulfil his planned transition of working in eSports. It was not that Dean desperately wanted to attend university, as he associated university with risk, rather he saw getting a degree as inextricably tied to getting a job, making taking on debt a necessary evil. Here, his parents' aforementioned perceptions of university ‐ “pushing” Dean “towards it” ‐ reflects a wider social belief that university degrees are translated into higher salaries in terms of a “better job”. It is not a moral judgement to suggest that if Dean were to go to university, he would likely be better positioned than his parents, whose household income is below the threshold for Dean to be eligible for free school meals.

This is something that Farah, another student at Woodhouse College, has experienced first‐hand. Farah was born in Iran but migrated to Rochdale in 2021 and, for a period, worked part‐time in a bakery before being laid‐off because she could not work during college hours. Her pursuit of university is framed by the fact that she could not convert her Level 3 diploma into employment as she has struggled to find work since leaving the bakery.

Farah If I don't go to university, I did get distinctions in my grades, but with Art and Design Extended Diploma degree, I cannot work anywhere. So that's why I have like a higher degree so that there will be something interesting in my CV. Till I finish all my studies in university, I won't be able to find a really great part‐time job because not even a take‐away will accept me.

Irrespective of the cost, Farah sees university as necessary to find employment within a crowded labour market, the opposite assumption made by Bilal and Arif. The spectre of debt still hangs over her head, as she says, “I do feel scared. They [teachers] told us you have to pay it back for 40 years of your life! I don't want to be in long debts or anything”. Farah is in the process of applying to university whilst studying a 1 year Level 2 Diploma in hairdressing, as she believed that this would enable her to find part‐time work in hair salons when she goes to university to help mitigate excessive levels of debt, as well as operating as a fall‐back option if she cannot convert her degree into a job, “I see every week people put adverts out wanting hairdressers”. Studying for a diploma in hairdressing is a risk management strategy Farah deploys against the uncertainty associated with a university degree. In a sense, this echoes Harrison's finding that young people see university as a form of insurance, as not going to university is seen as a risk within a localised labour market predicated on insecurity and in a context where a degree is increasingly a minimum barrier to entry for employment (Harrison 2018). The risk of getting a university degree is huge debt and no job. The risk of not getting a degree could be condemnation to the “precariat” (Standing 2014) as a variety of jobs now require a degree as a minimum barrier to entry. Here, there are regional inflections to these processes, as students are responding to their personal experience of local labour market conditions (their inability to find work), as well as their parents' experiences of the labour market; in Dean's case, his mother is unemployed whilst his Dad works in a snooker hall, and in Farah's case, her mother is unemployed whilst her Dad works in temporary contract employment in local warehouses, the sort of work Ben Rogaly has described as regionally specific (in the context of Peterborough) that is often deemed “foreigners work” due to a lack of protections and the undesirability (Rogaly 2020).

Perceptions of debt also figured prominently in London. Saffiyah and Iman, two working‐class black students enroled at Park Walk College, planned to attend university but shared similar concerns about debt. As a point of departure from Dean or Farah, both had part‐time jobs working “20‐30” hours a week in retail, reflecting different labour market opportunities for young people in London. Importantly, like Farah, neither student had a British passport, reflecting how precarious citizenship status is increasingly commonplace for migrants, refugees, and their children, many of whom traditionally settled in London (Rienzo and Vargas‐Silva 2022). These realities frame their perceptions of their transitions out of college, with both discussing university in‐depth as they sought ways to avoid debt, as their accounts were similarly characterised by “debt aversion”.

Saffiyah University is in my plan I guess, but I'm open to degree apprenticeships as well. Because I feel like university is a lot of debt. I know you're investing in yourself and that, but if I could try and get something that's just as good and I wouldn't have to pay, why wouldn't you want to do that? But I'd like to get into investment banking.

Iman I definitely want to go to uni, but probably not in the UK. I was thinking of going to Sweden because you don't have to pay for university. Like, I don't want to go here and just be in debt until I die. That's not it. So I would go Sweden, and also my Dad is Swedish, so it would be easy for me to get a citizenship. So then I was going to do English or like Sociology, History, something, as my undergrad and then I would do a teaching degree and become a teacher, but obviously I wouldn't teach in the UK either… I would probably do teaching. I would probably go to like, Kuwait or like, an Arab country or like anywhere else, and then you get a free flight back to the UK, so it's like, yeah.

Saffiyah and Iman approached university as a carefully considered financial decision that carries significant risk. For Saffiyah, this involved considering a very competitive city apprenticeship, and for Iman, it involved strategising to attend university in Sweden (which would be free) and then becoming a teacher abroad. Saffiyah's appraisal of university as, on the one hand bringing “a lot of debt” but on the other constituting “investing in yourself,” demonstrates the decision‐making process at play. To a large extent then, the fact that Saffiyah posits taking on such a huge financial burden as “investing” in herself, underlines the way that education has become commodified in ways that reinforce class divisions and shift how class inequalities are experienced and narrated by young people, many of whom digest dominant narratives of entrepreneurship and self‐improvement, here through university.^2^ This chimes with Harris and colleagues' findings, who identified how “the would‐be student is positioned as an economic subject driven only by an assumed need to maximise their financial self” (Harris et al. 2020, 133), as their respondents similarly discussed potential debt through the prism of investment and risk versus reward, which have proliferated since the 2007‐2008 financial crisis (Harrison 2018).

The fees and the perceptions around debt meant that Saffiyah would only consider attending university in London, as she later reflected, “I'd have to live at home to save on rent”. Attending university in faraway towns and cities is increasingly the preserve of the middle classes, which also has a racial dimension, as ethnic minority students are reluctant to study in areas thought to be less cosmopolitan than London (Gamsu et al. 2019). Tellingly, Saffiyah did not mention the apprenticeship again after this initial point, as she talked in greater depth about going to university to study something “economics‐based”, as both Saffiyah and Iman reluctantly saw university as the obvious route out of college. Hence, Saffiyah's initial statement, “University is my plan, I guess.” Here, Saffiyah's ability to draw upon investment banking, and later in the interview to name “J.P Morgan” and “Morgan Stanley” as two future career destinations, reflects the role of place in framing student perceptions of the future, as Saffiyah is invoking a career pathway that is inherent to London. No students in Morecambe or Rochdale could name a career pathway like this, nor could they invoke the name of two companies like J.P Morgan or Morgan Stanley, two investment banks with headquarters in Canary Wharf. It is worth reiterating the aforementioned point that investment banks operate essential class‐ceilings, favouring those from elite backgrounds (Ingram et al. 2023) who possess the requisite social and cultural capital to appear a good fit (Ashley and Empson 2017), making this a very difficult industry for Saffiyah to potentially break into.

Though Dean, Farah, Iman and Saffiyah were concerned about debt, other students believed the potential rewards outweighed the risks. For instance, Eloise, a catering student at Woodhouse College in Rochdale, argued that the repayments on any debt would be outweighed by future earning power.

Eloise Because I feel like university can give you a lot, and I think if I can get a good degree, I'll get more possibilities, because then I want to work myself quite high up… My mum laughs about how much debt I'm gonna be in, but like, she finds it a bit funny. But the thing is, it doesn't really matter, because once you earn enough, it'll be like five pound a month.

Part of the decision for Eloise is that she has no real knowledge about how student loan repayments work, suggesting that it is a cursory amount each month ‐ “it'll be like five pound a month” ‐ which is not true and reflects a lack of knowledge about how university works. Regardless, Eloise is apathetic about the potential debt she will incur, believing that going to university will result in higher earnings, making any debt worthwhile. Such misrecognition (see Callender and Davis 2024) of how the loan system functioned was common‐place at Newgate College, Rochdale's sixth form college, where students take A‐Levels and often transition to university. These A‐Level students strongly believed that attending university would lead to a “career”, a stand‐in for middle‐class professionalised employment associated with solid wages and upward progression (Singh 2024). As Neil, a first‐generation student on free school meals who had offers to attend elite universities, reflected, “The trade‐off is worth it because of how the loans work. It's more like a tax on the salary. And the trade‐off is a higher salary.” Ayesha, a British‐Pakistani working‐class student on free school meals, was similarly undeterred by the cost of university, reflecting to me, “It's covered by the loan, so I don't think about it much.” It is worth stressing that the graduate premium does tend to be higher for those who attend Russell Group universities, though the high concentration of graduate jobs in London, would likely necessstiate a prohibitively costly out‐migration to London to find the sort of careers these young people spoke of (see Ingram et al. 2023; Xu 2023; Yu et al. 2024) given how the UK economy continues to be orientated around the capital.

Still, the belief that a degree will translate into a graduate job reflects the “symbolic violence” outlined by Harris et al. (2020), which the university loan system is predicated on; the assumption that the debt is an investment in a promised future of gainful employment. The prospect of taking on debt is tolerated as a necessary part of ascending to university and, implicitly, experiencing upward social mobility vis‐a‐vis graduate employment. At the same time, Dean, Farah, Iman and Saffiyah were more aware of the unfair nature of the loans, hence their misgivings about debt and their attempts at mitigating against debt through various strategies; including part‐time work, studying abroad and a degree apprenticeship. Their reckoning with debt chimed more with Callender and Davis framing of debt as “strucutral violence” rather than “symbolic violence” (2024), in that these young people actively recognised the potential harms associated with the loan systems (rather than misrecognising), even if they expressed that they could not challenge it other than by shunning university, as Bilal and Arif did. Unlike those in Callender and Davis' study, these students have yet to attend university, let alone graduate, so have not been confronted with repaying the loans, so are not in a position to fully question the utility of the loans or reckon with any potential “false promises” (Ibid; pp. 954).

### 2.2 Migration and the Perceived Lack of Alternatives

Within the resurgent policy focus on working‐class attainment, much is made of how ethnic minority students are attending university at higher rates than their white counterparts (Sewell et al. 2021; Gov 2021), which is not a new observation, as ethnic minority populations have long been over‐represented at university (see Mirza 1992; Modood 2004; Alexander and Shankley 2020). Here, university is used as a stand‐in for social mobility, which ignores how ethnic minority students have higher dropout rates, are less likely to attend the most prestigious universities, are less likely to receive a good degree classification (two to one or above) and earn less in the labour market (Boliver 2017; Reay 2017; Alexander and Shankley 2020), even accounting for geography (Yu et al. 2024). It also obscures some of the factors behind why ethnic minorities attend university at disproportionate rates, which this section draws out.

Ana is a free school meals student in Rochdale who arrived in the town aged seven from Georgia. Her Mum does not work, and her Dad works in a local factory, having worked as a teacher in Georgia before the family migrated, which entailed earning a university degree. Such downward social mobility is common for migrants entering the UK, who find their qualifications undervalued, often experience xenophobia and racism in the workplace and in turn are forced to work non‐graduate jobs (see also Reay 2017; SMC 2020 on downward mobility). For Modood, writing in 2004, these processes “meant that not only did many [migrants] value education more than their white work mates but saw it as part of the process of reversing the initial downward mobility, especially in the lives of their children” (Modood 2004, 93). These dynamics were inherent within Ana's narration of why she wanted to attend university.

Ana My parents always told me “you can't not go to university”. I share the same values as them, that education is really important. They think that a degree is very important.

Amit For what?

Ana Just carrying you throughout life. They just feel like it gives you more chance of earning more money and everything.

To a large extent, Ana positions going to university in relation to her parents' sacrifices of migrating to Rochdale, which is a sacrifice she believes she should repay by fulfilling their desire for her to attend university (see Reay 2017). This is also correlated to the fact that many migrants, such as Ana, whose parents performed a downward class mobility, still possess cultural capital, in terms of a high valuation of education.

Sam, a Nigerian student who migrated to London just prior to beginning his A‐Levels, offers a similar account. His parents were university‐educated in Nigeria and performed a downward mobility on coming to London, as Sam now lives in a deprived London borough where they work non‐graduate jobs locally. His mother is a carer and his Dad is a security guard.

Sam I've been raised to see university as maybe the only option. Because in my culture, especially people in my ethnic group, they all go to universities. Mostly we're known, in Nigeria we're mostly known as the people that go for higher education, so, university is super important.

These cultural assumptions, combined with his parent's university backgrounds, shape Sam's valuation of education, as he has gained a place at a top London university to study engineering. Here, we see how migrants such as Ana and Sam possess cultural capital ‐ ways of thinking about education and a familiarity with university as a destination ‐ that aid their transitions out of college and are directly framed by immediate experiences of migration, reminding us of how class is material (and both live in precarious conditions) but is also composed of, in Bourdesian terms, dispositions and sensibilities (ways of thinking, feeling and acting) that are not exclusively tied to economic conditions. This is a point Modood alludes to, drawing upon Bourdieu, in relation to Pakistani attendance at university, there are different forms of capital so that it is possible for a family to be poor in oneform and rich in another, which fits with the case of socio‐economically disadvantaged Pakistani households having another kind of resource from which they can produce graduates (Modood 2004, 97). Crucially, Ana and Sam have knowledge of ‐ and familiarity with ‐ university that is passed onto them by their parents, which was not the case for many other students introduced in this paper.

Iman and Saffiyah in London expressed a similar rationale behind wanting to attend university. Though neither had parents who had been to university, they similarly expressed a desire to attend as framed by familial histories of migration.

Iman We're daughters of immigrant families, and there's lots of pressure on us, especially in this country, so we have to kind of do well. The whole reason that our parents came here is so that we could have a better life. So I guess there's all that pressure.

Saffiyah Yeah, I mean I guess the same. If I was to go university I'd be kind of doing it for my family as well… I think they kind of came to this country so for them kind of expect you… as you're their child for you to go to uni like it's kind of part of the deal. I brought up the apprenticeship idea and my Dad was so against it. He was like “you have to go university”. The older generation feel that university is like the key to life.

Iman My parents didn't have an education. My mum and my dad didn't have an education at school. So that's why they really want me to go into uni, they really want me to basically do what they never got the chance to do.

Like Ana, both suggest they must attend university to repay their parents' sacrifices in coming to the UK, essentially ruling out any non‐university pathways out of college. This is tied to taking advantage of educational opportunities that their parents did not have access to, reflecting what Heidi Mirza has described as the “migrant effect”, wherein migrants “pursue the goal of upward occupational mobility, particularly for the next generation, by striving for educational achievement and qualifications” (Mirza 1992, 172). Mirza coined the “migrant effect” in the early 1990s. Today, upward mobility via university is not as attainable, not least because the rapid expansion of universities means that more and more young people are funnelled into university, making it increasingly important to choose the right degree at the right university to secure post‐university employment within a shifting labour market, and what Brown and James refer to as increasing “job scarcity” (Brown and James 2020, 4). Resultantly, middle and upper‐class students, who are best equipped to respond to changing conditions, are able to maintain their relative dominance through the transmission of social and cultural capital, which are deployed to construct advantage as they can be converted into internships, work experience, relevant extracurricular activities and so‐on, all of which give them significant advantages in the graduate labour market (see Bathmaker et al. 2013; Reay 2017; Abrahams 2017). There is a racialised dimension to the deployment of such capitals, which are often read as white and middle‐class (see Singh 2021), which, in turn, disadvantages non‐white students and working‐class students, who will find it difficult to embody such dispositions or appear as the right “fit” within interviews for competitive graduate jobs (Rivera 2015).

Regardless, the pull towards university is pervasive and mediates these young people's perceptions of university, as does a lack of perceived alternative routes into work, rendering university their only tangible option out of college.

Ana I think especially just because I don't have connections here like my parents don't have connections. If maybe like Dad owned a business I’d go into that. I know a lot of people have parents to connect them with things. I have some female friends that like their mums are technicians and like beauty stuff and they’re going into that, but my parents don't have any of that… I don't have those connections. So for me it's like universities kind of feels like the only way.

This is an important explanatory factor into why so many children of migrants are inclined to pursue university, as they see few alternative routes into work as a result of a lack of relevant social networks in addition to wider societal xenophobia and racism (Battu et al. 2011), yet this is routinely ignored within wider discussions around the aspiration and educational attainment of migrants and ethnic minorities. Within Paul Willis' aforementioned Learning to Labour (Willis 1977/1981), it is notable that all of his respondents were white and boys, having pre‐ordained routes into work that ethnic minorities and women did not have access to because of wider racism and sexist attitudes, a theme Willis touched upon without exploring in great depth. Without social networks ‐ social capital ‐ to draw upon to find work locally, Ana is left with few options but to pursue a degree. Here, it is worth noting that Bilal and Arif (mentioned earlier) shunned university partly because they had locally available routes into work as barbers in Rochdale, as both secured part‐time jobs as barbers at Pakistani‐run barbershops in the area. Yet, this was against the wishes of their parents who believed university would be a better long‐term option for both. Though outside the scope of this paper, we can think of Bilal and Arif (as well as the other barbers interviewed in the wider project) as falling into barbering due to its local visibility in Rochdale as an ‘Asian thing’ (something reflected by staff at the college as well as students). Barbering though, represented an alternative to university that enabled them to make money in the short‐term.

Absent such obvious local routes (which are also precarious), Saffiyah and Iman likewise experienced a desire for alternatives, partially framed by the prospect of taking on excessive debt.

Iman I wish there was other options. I wish there was another route, and I could still live, like, a good quality of life. I also feel because I'm not as privileged as some other people in society, I have less options for myself. I can't take as much risk as maybe, let's say, a white boy that comes from a middle class family. I feel like I'm a bit more restricted on what I can do. There's more pressure ’cause like me being a black girl, it's definitely gonna be harder for me to get on in life than say if I was a white guy that came from like a wealthier family.

Saffiyah Definitely. It's facts. I think a lot of people that I had some friends from previous schools that were from like middle, upper classes and they can take risks and like they don't really care if they don't do well in school that much because at the end of the day they've got their family to go back on to which are wealthy whereas for someone that's not from a wealthy family it's a thing where I can't really take that risk of just being careless and just doing what I want to do.

Like Ana, Saffiyah and Iman situate university ‐ to an extent ‐ as being their only option in the absence of viable alternatives, which in part is framed by their positionality as the child of a migrant in Iman's case and as a migrant in Saffiyah and Ana's. As such, Iman and Saffiyah bemoan a lack of alternative routes out of college but believe that any alternative plan would constitute a risk that they cannot afford to take. University then, regardless of the excessive fees and the associated debt, is understood as less of a risk than not going. These realities extensively shape their aspirations, ruling out pursuits of passion or leisure that necessitate risk‐taking. This contrasts with how middle and upper‐class students are encouraged to pursue their interests and passions unabashed, as captured in Shamus Khan's study of students at the elite US boarding school St Paul's, who are conditioned “to think of the world as a space of possibility within which they could realise their interests” (Khan 2012, 67).

### 2.3 Pursuit of Dignity and Respect

A major theme uniting my respondents applying for university was the pursuit of dignity. To have dignity is to have self‐worth, but more to be treated with respect and be recognised by others as a person of value (Sayer 2007). Through no fault of their own, many are ultimately not recognised as worthwhile, which extends beyond race and class to gender, sexuality and who is considered able‐bodied or not (Sayer 2007; Skeggs 1997; Mustaffa and Dawson 2021). Dignity can be understood in relation to its opposites: indignity, shame, stigmatisation, and disrespect, which bring low self‐esteem, shame and humiliation (Sayer 2011; Tyler 2020), which reflects Sennett and Cobb's “hidden injuries of class” (Sennett and Cobb 2023). The working‐class people interviewed by Sennett and Cobb in 1970s Boston sought dignity by escaping their class position in ways that were often tied to education, either for themselves or their children (Sennett and Cobb 2023). This reflects the distinctions made between those who are university‐educated and those who are not, a distinction between the supposed worth of people. Here, it is worth saying that working‐class people have historically been denied access to education, excluded from certain educational institutions and so‐on. Yet, university and the pursuit of education has never been exclusively the preserve of the middle‐class (see Jonathan Rose 2001, Johnson 1979). Though today education is increasingly commodified, presented as a “risk versus reward” calculation or investment, education does still have emancipatory potential that is desirable for working‐class youth (Freire 2017) who seek out dignity, knowledge, the chance to pursue a passion and to accomplish something otherwise denied.

The young people I met saw university as a route to achieving dignity, framed as being recognised by others and treated with respect. As predominantly first‐generation they spoke about wanting to make their parents proud by achieving something their parents did not. The following excerpts from Abi, a white working‐class student in Rochdale, Eloise, Dean, and Tim, a white working‐class student in Morecambe, one of only two students I met at MTC with aspirations to attend university.

Eloise They're very happy about it because none of them went to university. So they're like, it's a new opportunity for me to tell them about as well.

Abi They're very happy that I've decided to go to university because they didn't go and so they're like it's very important that you go to, you get your education while you can.

Dean My parents are very proud because I'm actually the first in my family to finish college and so the first to go to university.

Tim They think it's amazing, they're happy for me. They’re proud. They said if I didn't want to, that's obviously fine, I can do what I want, but they're happy that I'm doing it.

Going to university as first‐generation students is a source of pride for the students and their parents, demonstrating the affective dimensions of class and the cultural appeal of universities beyond the supposed graduate premium. Until the 1990s and the expansion of higher education provision, university was not the normative transition out of education for working‐class young people, many of whom were denied opportunities to enter what were ostensibly middle‐class institutions. In part, this reflects why their parents had not had the chance to attend university, and why, relatedly, parents saw university as potentially offering a better life to their children in the form of upward social mobility. Framing university in this way is not dissimilar to the “migrant effect”, outlined in the previous section, which likely speaks to a lack of alternative routes into work for working‐class youth in the aftermath of the 2007‐08 financial crash and wider processes of deindustrialisation. Ayesha, a British‐Pakistani student on free school meals at Newgate College who was introduced earlier, similarly spoke to the cultural pull of university.

Amit Did you ever consider any other routes, other than A‐Levels?

Ayesha I think I was just more interested in like doing A‐levels and going to university, getting a degree, like that kind of route. I wasn't really interested in like apprenticeships and other stuff like that…. because my parents don't have degrees or anything so I always wanted, like I want to go to uni and get a degree because I know they haven't got it and I think they would be proud to see me get one too.

On the one hand, Ayesha constantly involved the idea of securing a “career” (Singh 2024) as a motivating factor behind her desire to go to university, coded as professional middle‐class employment, but on the other, she repeatedly referenced fulfilling something her parents were unable to do. As a result, Ayesha ruled out any alternative routes into work, offering a conflicting account from Ana, Saiffyah and Iman in the previous section, as she never wanted to pursue any other route than university, as a means of making her parents proud. Intuitively, pride, dignity and respect were also recurrent themes for Saffiyah and Iman in London, demonstrating their universal pull across racial and ethnic groups and across geographic locations.

Amit You both work now, did you ever consider just staying on doing that?

Saffiyah No! I want a career, I want a career. Like, honestly, I have respect for people that can do that for their whole life. Because I feel like most people, from what I've noticed, it's not usually a choice. It's usually they have to. A lot of people I know work them jobs tend to have just come to this country or something like that. But there's no security in that, like long term where do you go?

Amit Do you think it's important to have a career?

Saffiyah Yeah yeah I really want a career.

Iman Yeah. I just want to be like accomplished like I was very literate. I want my family to be proud, like when I have kids for them to say “mum is so accomplished and literate.”

A desire to attend university is not solely about material gain, but is intertwined with a sense of self worth and pride for themselves and for their families, who, as noted, they feel they must repay. In addition, both link university to “a career”, which is presented as professional middle‐class employment which will allow them to feel “accomplished” and “literate”, traits often situated as antithetical to being black and working‐class. We might consider how the pursuit of dignity is mediated by experiences of racism, as migrants and the children of migrants are routinely cast as an undesirable underclass who are treated with suspicion and therefore denied dignity and respect. For Saffiyah and Iman, the allure of being regarded as “accomplished” and “literate” is staking a claim to dignity and respect that is routinely denied to working‐class subjects (see Skeggs 1997), with notions of respectability produced in relation to racial classifications within the imperial project (Mosse 2020). Again, this speaks to the emancipatory potential of education, wherein Iman and Saffiyah are concerned beyond a “career” alone, as a large part of the appeal is about being deemed a person of value. As Mustafa and Dawson argue, “Education self‐dignifies and humanises the abjectly despised and discarded” (2023; pp. 9), it provides a chance to imagine oneself as a person of value and the chance to be recognised by others, which is central to notions of dignity (Sayer 2005).

In addition, it is worth noting that Saffiyah and Iman rule out the prospect of localised menial work in contrast to Leon and Maria (mentioned earlier), who ruled out the prospect of going to university, believing that their transition to work would involve working locally in “pubs” or “cafes”.” Though in London such jobs might be dominated by a migrant underclass, in Morecambe, that is not the case. Localised, insecure, menial jobs are a visible local option for young people. As such Maria, who ruled out attending university because of perceptions of debt, is considering completing a Level 3 course on completion of her current course, or finding work in a local pub; “I'm kind of between the two now… probably Level 3. Or try and make some cash I guess, that's why I said pub maybe”. Ultimately, this is about the spatial divisions of labour (Massey 1979); in London graduate jobs are visible, in Morecambe they are not (or at least not obviously for working‐class young people), making university a less obvious transition for working‐class young people such as Maria or Liam (see also Davies and Donnelly 2023). It is relevant that 22 out of 24 young people in Morecambe could not meaningfully envisage going to university, so dignity or access to it is spatially distributed.

Here, I turn to Denis, an A‐level student in London who was born in Bulgaria and came to the UK aged 16 to work in construction with his father, with the view to training to become a plumber, a trade associated with stability and good wages. However, Denis eventually abandoned his pursuit of plumbing to study A‐Levels at Park Walk College (after completing a year‐long GCSE package course), having since applied to Russell Group universities to study Business Management.

Amit Why did you change your mind about plumbing?

Denis I want to use my brain. I was like, I have some potential, might as well try, you know. I can always go back.

Amit Why did you think that was important though, to do something with your brain?

Denis uh… Even at the time I was like… it's just more interesting, just more interesting. At the time I already had experience in working as a laborer, and that was not interesting, not fun at all. I was like, you know, you have to do something you have to work your brain, you're gonna still getting tired and all that it's different kind of tired…

It was not so much that Denis felt these jobs were beneath him; his cousin works in plumbing, and his dad works in construction, which he also spent time doing. Yet, Denis distinguishes between different forms of labour, seeing himself as having “potential” in opposition to other people who funnel into the trades. He knows that plumbers earn more than most white‐collar graduates in the context of an ever‐diminishing graduate premium. Still, this does not matter for Denis because using his brain is a source of pride, bringing respect not afforded to the plumber. Once more, we see how the moral and cultural dimensions of class shape the aspirations of young people in ways that transcend material gain. He went on to say, “I was thinking, I can do more. Plumbing's not really like, a highly… [*Pauses*] You don't need to be that intelligent to be a plumber.” As Richard Sennett reflects in the 2023 introduction to *Hidden Injuries of Class*, aptly comparing plumbers and business management graduates; “white‐collar work has greater prestige than manual work, but plumbers are more likely to make more, and prosper longer‐term, than… kids fresh out of university armed with business management degrees” (Sennett and Cobb 2023; pp. xi). Much cultural work has been done to distinguish between the educated and uneducated, which frames young people's aspirations beyond notions of a “graduate premium”. Savage found similar in his assessment of the Mass Observation directive where respondents distinguished “between brainwork and manual work” in discussing class, as he notes, “The idea that the middle classes worked with their brains, and hence were more intellectual, cultivated and superior to the working class runs very deep for many of the Mass‐Observers” (Savage 2007). The perceived value of going to university outweighed the fact that he might have earned more as a plumber, which he observed later on in our interview, telling me, “If I wanted just money, I would have stayed plumbing, I would have continued. So money wasn't the motivation there.” There is a wider point to be made here about how university, as commodified as it is, is not just about the pursuit of a career, but is also tied up with a desire to learn, intellectual stimulation and fulfillment, which also came through from other students. Here, we can think about how the student loan then opens up the opportunity to live dignified lives, which does involve a certain form of work, but also allows working‐class young people to feel valued. Writing in the context of debt and racial capitalism, Mustafa and Dawson argue that “Black people may value student loans as a rare opportunity to access the invaluable—the promises of a full livelihood for the credentialled” (Mustaffa and Dawson 2021, 9). We can extrapolate this out to working‐class young people across racial groups, gender and geographic location. Beyond considering experiences of class and inequality, we must remember that people are constituted as subjects with feelings, desires, aspirations and passions, which in this case include desiring to attend university as a means of making others proud and feeling like a person of value.

## 3 Conclusion

This paper sought to foreground how working‐class young people in London, Rochdale and Morecambe make sense of attending university in the context of soaring university fees and significant changes in the labour market. Of the 60 students involved in the wider project, 28 aspired to attend university, which speaks to the continued appeal of university as the transition out of education and into work is prolonged. This partially reflects how many jobs require a degree as a minimum barrier to entry in an overly crowded labour market. It also reflected a lack of clear alternatives, particularly for those who are not pursuing a trade, as they would then be faced with local menial work that is precarious and undervalued. This is largely the type of work their parents did (if they were in work), who, in turn, encouraged their children to pursue university as a means for a better life. Students then find themselves caught in a bind; either pursue an expensive and risky university degree, that might bring with it a higher salary, though, as stated, the graduate premium is unevenly distributed geographically, which has implications for all of the young people I spoke to, including those from a deprived East London borough (Yu et al. 2024). Or do not go to university and risk falling into the “precariat”, taking on low‐paid, insecure work within the local service sector, work many of them have experience of doing. Here, part of the decision‐making reflects Harrison's assertions that university can act as expensive “insurance” against labour market exclusion (Harrison 2018).

University was then imagined as a carefully considered “risk versus reward” calculation, in ways that would not be the case for middle or upper‐class students, who go to good universities and get graduate jobs with far less friction (see Ingram et al. 2023). The reality of student debt certainly figured into student plans, even if many continued to pursue university against the aforementioned lack of alternatives. In part, university was bound up with a common pursuit of dignity, as working‐class young people wanted to make their parents proud and be treated with respect, as people of social value. This reflects the continued cultural pull of university and of a university degree as being bound up with notions of dignity (Sennett and Cobb 2023), that can override material realities. For Denis, who had an alternative route into work as a plumber, this involved him shunning higher wages due to his desire to use his “brain” as opposed to his hands to make a living due to the higher social value he associated with a graduate job relative to blue‐collar work.

However, in the context of university's increasing commodification, and the structural and symbolic violence associated with the student loan system (Harris et al. 2020; Callender and Davis 2024), it is difficult to know whether those who are planning to attend university will acquire the dignity they seek. On the one hand, a degree is still associated with social value, pride and the ability to see oneself as person of value, as someone who can repay familial sacrifices at a cultural and material level. On the other hand, the failure to convert a degree into meaningful graduate employment, translated into earning power, can result in the internalisation of narratives of personal failure, particularly if likely detached from wider structural critiques of a commodified education system and an unmeritocratic labour market context (see e.g., Friedman and Laurison 2019). Such a context is concerning for many of the young people I met, given the aforementioned spatial dynamics of the graduate labour market and the necessity to deploy various capitals in order to access graduate employment. Can university in this context ever be dignifying in totality? Within this commodified landscape, the best‐case scenario is that university will be an enriching experience that also results in individual experiences of mobility. At worst, university can compound experiences of shame and indignity, not least if students fail to convert degrees into graduate employment. True humanisation via education is not likely within a system predicated on winners and losers.

## Data Availability

The data that support the findings of this study are available on request from the corresponding author. The data are not publicly available due to privacy or ethical restrictions.

## Endnotes

^1^

https://explore‐education‐statistics.service.gov.uk/find‐statistics/participation‐measures‐in‐higher‐education/2022‐23.

^2^
This is a point made clear by Reviewer 1!.
